# Behavioral Phenotyping of Juvenile Long-Evans and Sprague-Dawley Rats: Implications for Preclinical Models of Autism Spectrum Disorders

**DOI:** 10.1371/journal.pone.0158150

**Published:** 2016-06-28

**Authors:** Katherine M. Ku, Ruth K. Weir, Jill L. Silverman, Robert F. Berman, Melissa D. Bauman

**Affiliations:** 1 Department of Psychiatry and Behavioral Sciences, University of California, Davis, Sacramento, California, United States of America; 2 Department of Neurological Surgery, University of California, Davis, Davis, California, United States of America; 3 The MIND Institute, University of California, Davis, Sacramento, California, United States of America; 4 California National Primate Research Center, Davis, California, United States of America; University of Lethbridge, CANADA

## Abstract

The laboratory rat is emerging as an attractive preclinical animal model of autism spectrum disorder (ASD), allowing investigators to explore genetic, environmental and pharmacological manipulations in a species exhibiting complex, reciprocal social behavior. The present study was carried out to compare two commonly used strains of laboratory rats, Sprague-Dawley (SD) and Long-Evans (LE), between the ages of postnatal day (PND) 26–56 using high-throughput behavioral phenotyping tools commonly used in mouse models of ASD that we have adapted for use in rats. We detected few differences between young SD and LE strains on standard assays of exploration, sensorimotor gating, anxiety, repetitive behaviors, and learning. Both SD and LE strains also demonstrated sociability in the 3-chamber social approach test as indexed by spending more time in the social chamber with a constrained age/strain/sex matched novel partner than in an identical chamber without a partner. Pronounced differences between the two strains were, however, detected when the rats were allowed to freely interact with a novel partner in the social dyad paradigm. The SD rats in this particular testing paradigm engaged in play more frequently and for longer durations than the LE rats at both juvenile and young adult developmental time points. Results from this study that are particularly relevant for developing preclinical ASD models in rats are threefold: (i) commonly utilized strains exhibit unique patterns of social interactions, including strain-specific play behaviors, (ii) the testing environment may profoundly influence the expression of strain-specific social behavior and (iii) simple, automated measures of sociability may not capture the complexities of rat social interactions.

## Introduction

Autism spectrum disorder (ASD) is a neurodevelopmental disorder characterized by impairments in social interactions and stereotyped behaviors [1]. Given the prevalence and societal impact of ASD, there is an urgent need for preclinical research to evaluate potential causes, determine the underlying neurobiology and discover novel therapeutic interventions [2]. Developing valid animal models and novel treatment targets has proven exceptionally challenging for complex brain disorders, such as ASD, where the varied symptoms are difficult to model in a nonhuman species [3–6]. The majority of ASD preclinical research has been carried out in mouse models that utilize standardized behavioral phenotyping tools to measure ASD-relevant deficits in social behavior, screen for repetitive behaviors and restricted interests, and evaluate associated symptoms, such as anxiety [7]. Although standardized approaches have led to more coordinated preclinical research efforts, there remains a need to develop additional behavioral tests that capture the full spectrum of social deficits relevant to ASD [8]. Here we explore the use of the laboratory rat (*Rattus norvegicus*) as a model system that can be used to improve translational potential of ASD preclinical research efforts [9].

The laboratory rat has been the biomedical species of choice for testing drug efficacy, dosage and toxicology for preclinical research, and may be particularly well-suited for ASD focused research. Rats demonstrate enhanced cognitive abilities and a rich social repertoire paired with neural complexity, particularly in brain regions implicated in ASD pathology such as the frontal cortex [10–12]. The rat social repertoire includes a prolonged period of juvenile play that may provide a preclinical model system to explore social impairments in more detail than is possible with other rodent models [13,14]. The potential of the rat ASD model has been established in prenatal toxicology studies with valproate [15–17], and is rapidly expanding to explore genetic susceptibility [18,19], underlying neurobiology [20] and potential therapeutic interventions for ASD [21–23]. Although there is increasing interest in utilizing the laboratory rat as a preclinical tool for ASD research, the behavioral outcome measures that are employed in rat ASD models are highly variable and there is a clear need to establish a robust and replicable rat behavioral testing battery that is relevant to core and associated symptoms of ASD [24,25].

The aim of the present study was to merge the rich literature of rat social behavior studies [26–33], with the demand for high-throughput behavioral assays amenable to preclinical ASD models [34]. We elected to establish our behavioral testing approach in two commonly used strains of laboratory rats—the albino Sprague-Dawley (SD) and pigmented Long-Evans (LE) that exhibit strain specific patterns of behavior [35–39]. Numerous studies have documented differences in social interactions among rat strains [40,41]. In general, SD rats are described as having robust baseline levels of play behaviors [42,43], though the “turn and face” play behavior characteristic of LE rats may provide an opportunity to quantify more nuanced social interactions [26–33]. To capitalize on the complex repertoire of rat social development, we focused on behavioral assays targeting ASD-relevant impairments in social behavior. The strains were also compared using assays targeting ASD-relevant repetitive behaviors, as well as associated symptoms, such as anxiety/exploration (elevated plus maze, open field paradigm), sensorimotor gating (pre-pulse inhibition[PPI]) and learning and memory (Morris water maze) (Table 1) [7]. Duration of self-grooming bouts, were quantified as an index of repetitive or stereotyped behaviors [44]. We then utilized a modified version of the marble burying task, originally developed in mice to evaluate compulsive burying, but more recently adapted for use in rats [45,46]. Social interactions were initially quantified using a simple, automated three chamber-chambered social approach paradigm commonly used in mouse models of ASD [47]. In this paradigm, sociability is defined as spending more time in the chamber with the novel conspecific as compared to an identical chamber without a novel conspecific. We then carried out a more fine-grained assessment of unconstrained social interactions between age/strain/sex matched novel pairs (i.e., social dyad) at juvenile and young adult developmental ages.

**Table 1:** Test Order.

Age	Task
PND 26	Elevated Plus Maze
PND 27–28	Social Approach
PND 29	Open Field
PND 34–35	Prepulse Inhibition
PND 36–37	Juvenile Social Dyad
PND 40–51	Morris Water Maze
PND 54	Marble Burying
PND 55–56	Young Adult Social Dyad

There is a rich literature describing numerous approaches for quantifying juvenile rat social interactions [48–50]. We drew from this existing literature to develop a relatively high-throughput protocol designed to maximize translational potential with other preclinical ASD models, including both mouse and nonhuman primate [51,52] (Table 2). Given that play is a behavior common in many young mammalian species, including human children, juvenile monkeys and rats, we focused our efforts on quantification of play behaviors [53–55]. Play fighting is the most common form of play behavior in rats [56] and is initiated when one partner uses their snout to nuzzle the nape of the neck of the other animal and the partner, in turn, defends their nape from such attacks by rotating to its dorsal surface or evading the attacker. As compared to mice, rats exhibit more complex patterns of play fighting characterized by reciprocal bouts of attack, defense and counter attacks [26]. We first quantified the amount of time the age/sex/strain pair engaged in play (i.e., pouncing/playful nape attacks, pinning, and rapid chasing) versus social exploration (i.e., sniffing, following, non-play contact), social proximity (i.e., within 2cm but not playing or exploring) or nonsocial activities (i.e., self-grooming, cage exploration). We then incorporated a more fine-grained assessment of reciprocal play behaviors by quantifying the frequency of nape attacks initiated or received by the focal animal. Although the young SD and LE rats in the present study performed similarly on the majority of the behavioral assays, we did detect significant strain differences in the duration and frequency of play behaviors. The implications of these findings are discussed within the broader context of utilizing rats in preclinical ASD research.

**Table 2:** Social Dyad Ethogram.

Behavior	Definition
**Nonsocial Activity**	Rat is actively exploring the arena (sniffing, moving around)
**Social Exploration**	Rat is sniffing, following, contacting or grooming other rat.
**Social Play**	Rat displays play behavior (nape attack, pinning, pouncing, chasing)
**Social Proximity**	Rat is within 2 cm of the other rat, but not touching.
**Self-Grooming**	Rat is licking or scratching itself
**Nape Attack (Frequency only)**	Initiate = Focal rat is touching the partner’s nape with its snout. Receive = Focal rat’s nape is touched by the partner’s snout Mutual = Both initiate and receive definitions occur in rapid succession

## Methods

### Subjects

Male Sprague Dawley (*n* = 12) and Long-Evans rats (*n* = 12) were shipped from Harlan Laboratories at the time of weaning to the University of California at Davis on postnatal day 21 (PND 21). As ASD occurs in the 4:1 male to female ratio, the current study focused on establishing a behavioral battery for male juvenile rats. Future efforts will expand to include both sexes. Rats were given identification marks and weighed on PND 22. For social tasks, subject rats were paired with unfamiliar stimulus rats of the same strain, sex, and weight. The stimulus rats were shipped and weaned at the same time as the subject rats (8 SD and 8 LE). All rats were housed in same strain groups (subject rats were housed in pairs; stimulus rats were housed in groups of three) in a temperature and humidity controlled vivarium on a 12 h light-dark cycle. Rats had access to food and water ad libitum throughout experimentation. All testing was conducted during the light phase of the 12 h light-dark cycle. Standard housing consisted of polypropylene cages (30.5 cm x 35.6 cm x 20.3 cm) with a high top wire lids, cob bedding, and nesting. All rats were weighed weekly and handled for 2 minutes per day for three days prior to the onset of behavioral testing. Animals were euthanized following behavior assessment by carbon dioxide (CO_2_) asphyxiation. Death was confirmed by physical examination. This study was carried out in strict accordance with the recommendations and approval of UC Davis Institute of Animal Care and Use Committee.

### Behavior Experimental Methods

The effects of strain were characterized using a behavioral test battery that began at PND 26 and concluded at PND 55–56. Testing assessed a range of behaviors including exploration, sensorimotor gating, anxiety, repetitive behaviors, learning and sociability. Tests were conducted on separate days in the following order to reduce the influence of sequential testing: elevated plus maze, social approach, open field, pre-pulse inhibition (PPI), social dyad, Morris water maze, and marble burying (see Table 1). With the exception of PPI, all testing was conducted under dim illumination of 10–15 lux. Before each trial, the testing chambers were thoroughly cleaned and disinfected with 10% Nolvasan solution (Fort Dodge Animal Health, Fort Dodge, IA).

#### Elevated Plus Maze (EPM)

On postnatal day 26, subject rats performed an elevated plus maze task. The elevated plus maze is a black polypropylene plus-shaped platform consisting of two opposite enclosed arms (10 cm x 50 cm) and two opposite open arms (10 cm x 50 cm). The arms meet at a center square platform (10 cm x 10 cm). The enclosed arms are surrounded by 10 cm high walls. The entire maze is elevated 100 cm off the ground. EPM testing occurred between 9:00 and 14:00 hr. Rats were allowed to habituate to the test room conditions for five minutes. At the start of each trial, the test rat was placed on the open center platform facing an open arm. The rat was allowed to explore the apparatus for 5 minutes and was video-recorded using video-tracking software (Ethovision Version 4.0, Noldus Information Technology, Netherlands). The tracking software used the midpoint of the body of the rat to distinguish when the test subject had entered or exited an arm boundary. Trials were scored using two parameters in order to assess anxiety-like behavior: time spent in each arm and number of entries and exits for each arm. Other behaviors, such as head dipping and stretched attend postures were not scored in the present study [57–59], though videos for each subject were archived and facilitate future behavioral quantification.

#### Social Approach

Subject rats performed a social approach task on postnatal days 27–28. Stimulus rats were age/strain/sex/weight matched rats that were housed in the same vivarium, but had not previously interacted with the subject rat. Subject and stimulus rats were placed in a room of 13 lux illumination and allowed to acclimate to test room conditions for 5 minutes. The subject rat was then placed in a square, three-chambered box made of clear plastic (101.6 cm l x 101.6 cm w x 33.7 cm h) (Stoelting Co., Wood Dale, IL). Each chamber had dimensions of 101.6 cm l x 33.3 cm w. The subject rat was allowed to habituate to the empty three-chambered box for ten minutes with free access to all three chambers. Two plastic cylindrical cages (13.3 cm diameter x 21.0 cm h) were then placed in the left and right chambers. The cages consisted of a black circular plastic base and lid connected by clear plastic vertical rods spaced 1.27cm apart. The stimulus rat was placed under one of the cages in a side chamber while the other cage was left empty and placed in the opposite side chamber, serving as a novel object. The side chambers containing the stimulus rat and the novel object were alternated between left and right side chambers between individual subjects. Stimulus rats were the same strain as the test rats and had habituated to the enclosure prior to testing. At the start of each trial, the subject rat was placed in the middle of the center chamber facing the back of the arena and allowed to access to all three chambers for 10 minutes. Trials were recorded using video-tracking software (Ethovision Version 4.0, Noldus Information Technology, Netherlands) and were scored for focal observations using video scoring software (Observer Version XT12, Noldus information Technology, the Netherlands). Subject rats were scored on three parameters: time spent in each chamber, time spent in the immediate proximity (within 2cm) to the social or nonsocial cage, and number of entries into each chamber. Sociability was defined as spending more time in the chamber containing the stimulus rat than the chamber containing the novel object, and more time spent in the immediate proximity (2 cm) of the stimulus rat than the novel object.

#### Open Field

On postnatal day 29, subject rats were tested for spontaneous locomotor activity. Locomotion was measured using a fully automated contrast-sensitive video-tracking program (Integra Accuscan, Columbus, OH, USA). The set-up allowed the simultaneous tracking of four animals, using four separate square observation arenas (41.3 cm length x 41.3 cm width x 29.2 cm height). At the beginning of the trial, the subject was placed in the center of the arena. The sampling rate was set to five samples per second. Spontaneous activity was measured over a 60 minute period. Distance moved (cm) was calculated every 1 minute. Parameters used to measure subjects’ locomotion were the following: distance travelled, time spent in center of arena, horizontal activity, and vertical activity.

#### PPI

PPI testing occurred on postnatal days 34–35. Subjects were placed in a clear plastic cylinder, which was attached to a platform connected to piezoelectric transducers. The platform was placed in a sound-reducing chamber containing speakers and controlled using specialist software (SR-Lab, San Diego Instruments). Subjects were allowed to acclimate to the sound chamber for 5 minutes with a 65 dB background white noise level. Each session consisted of a pseudo-randomized presentation of 5 different trial types. The trial types were as follows: 120dB startle alone, 120dB startle with 74dB prepulse, 120dB startle with 82dB prepulse, 120dB startle with 90dB prepulse, no stimulus (white noise). Each trial type was presented in 10 blocks and was randomized within blocks. Pre-pulses at three different intensities (74, 82, 94dB) were played 120 ms prior to the startle pulse (120dB, 40 ms) to assess pre-pulse inhibition. The intertrial interval varied randomly between 10 s and 20 s. Percentage PPI was calculated using the following equation: PPI = [1 –(Prepulse/Max Startle)] x 100.

#### Social Dyad

Subjects were run in reverse order of the social approach task. Trials were conducted at two developmental time points: juvenile (PND 36 and PND 37) and young adult (PND 55 and PND 56). Stimulus partners were age/strain/sex/weight matched rats that were housed in the same vivarium, but had not previously interacted with the subject rat. Both subject and stimulus rats were placed into respective transfer cages and were isolated in a quiet dimly lit adjacent room for ten minutes while separated by a visual barrier. The apparatus consisted of three identical Plexiglas chambers (41.9 cm w x 29.2 cm h x 41.9 cm l), two side chambers used for acclimation and a center arena used to videotape dyad interactions between the subject and stimulus rat. The subject rat and stimulus rat were moved to test room of 12 lux illumination and placed in separate acclimation arenas and allowed to acclimate for 5 minutes. Between trials, subject and stimulus rats alternated between acclimating in the left or right arenas. Both rats were then placed into the center arena and video recorded using a Sony HDRCX240/B Video Camera with 2.7-Inch LCD (Black) fixed to a tripod. The rats were allowed to interact for 10 minutes. One LE rat was excluded from the analyses due to a failure to record the entire 10 minute session. Videos were scored using Observer XT12 software (Observer Version XT12, Noldus information Technology, the Netherlands). Focal observations were used to quantify the amount of time the subject rat spent in nonsocial activity (exploring the cage or self-grooming) and the amount of time spent in three broad categories of social behavior: (i) social play—a composite of well-characterized play behaviors including pouncing/playful nape attack, pinning, wrestling, boxing, tail pulling, and chasing, (ii) social exploration—a composite of social investigation (i.e., sniffing, following) or non-play contact (i.e., grooming, licking, crawling over, under) and (iii) social proximity—scored when the rats were within 2cm of each other, but not actively engaged in investigation, contact or play behaviors (Table 2).

Our definition of play was a composite of several behaviors described in rat play behavior literature [52], which included: 1) pouncing/nape attack-nuzzling the nape of the conspecific’s neck with the tip of the snout followed by a rubbing movement; 2) pinning—upon contact of the nape, the recipient animal fully rotates around the longitudinal axis of its body, ending in a supine position with the other subject standing over it; 3) boxing/wrestling—rearing in an upright position towards the other subject combined with rapidly pushing, pawing, and grabbing at each other, or wrapping around the other subject; 4) partial rotation—upon contact of the nape, the recipient animal begins to rotate along its longitudinal axis, but then stops and keeps one or both hind feet firmly planted on the ground and 5) Evasion/Chasing—upon solicitation, the recipient animal avoids contact with the nape by leaping, running, or turning away from the partner/moving or running forward in the direction of or pursuing the other subject, who moves away. Other components of the rat social repertoire, including social investigation and grooming, are not considered primarily related to play [24,25] and we scored under the category of “social exploration” that included a composite of several social investigation (sniffing, following) and contact behaviors (grooming, licking, crawling over, under). We distinguished chasing (defined as a more vigorous form of following characterized by close proximity and fast pace) from following (slower pace, more than one body length apart) and included the latter under the category of social exploration rather than play [41,60]. Although crawling over and under behavior is often a precursor to playful interactions [41], and has been scored under a separate category of play solicitation behaviors [57], we grouped these contact behaviors under the category of social exploration to more readily distinguish distinctive and unambiguous measures of play. We also included a third category of social proximity, defined as being within 2cm of the other animal, but not playing or investigating to measure the amount of time animals spend simply being proximate but not interacting.

Bouts of play fighting in rats have been described as beginning when one rat solicits another animal by attempting to nose or rub the nape of the neck (pouncing/nape attack) [29,56]. The animal that is pounced upon can then respond by rotating onto its dorsal surface (being pinned) or by evading, which often results in chasing by the soliciting rat. Although frame by frame analyses of response to nape attack provides the most comprehensive assessment of play interactions [30], here we sought to establish a high-throughput quantification of reciprocal play behaviors focusing initially on the frequency of nape attacks initiated and received by the subject rat. Nape attacks initiated and received in rapid succession within 1 second were scored as a separate category of mutually exchanged nape attacks. As all dyadic interactions were scored from video, more in depth analyses of response to the nape attack can be quantified from archived videos in the future.

#### Morris Water Maze

Subjects were tested in the Morris Water Maze starting at PND 40. Testing was conducted in a circular pool (diameter 157.38 cm, height 61.0 cm) filled with water (26°C) in a room of 10 lux illumination containing stable visual cues (blue tape on the wall, computers, laboratory equipment). The pool was divided into four quadrants, West, North, East, and South (A, B, C, D respectively). A video camera was located above the center of the maze and recorded the animal’s position in the pool. Rats were video-recorded and tracked using Ethovision software (Ethovision Version 4.0, Noldus Information Technology, Netherlands).

A cylindrical platform (Base = 20.3 cm. Diameter of cylindrical platform = 10.2 cm. Height = 125.7 cm) was placed in the middle of the North quadrant of the pool. The tank was then filled with water to a depth of 2 inches below the rim so that the platform was 1.0 cm below water level. Each animal received four trials (90s maximum) per day, over five consecutive days. Trials started from one of the four starting locations (N, S, E, W) in a pseudorandom fashion, with a given starting location occurring only once within each block of three trials. On each trial the rat was then placed in the designated starting position facing the rim, and allowed to swim until it located and mounted the platform or until a maximum of 90 seconds had expired. Rats finding the platform were allowed to stay there for 30 seconds. Rats that did find the platform were placed onto the platform and allowed to stay for 30 seconds. If the rat dove off the platform, it was placed again onto the platform for the remaining time. Between trials rats were placed on a towel in a Plexiglas cage warmed by a heating pad underneath.

Probe test (PND 45)—A probe trial was conducted on day 5 of testing. The platform was removed, and animals were placed in the pool facing the wall in quadrant opposite where the escape platform was located and allowed to swim for 90 s. Time spent in each quadrant, as well as number of times the rat cross the former location of the platform were recorded.

Platform reversal (PND 46–50)—For reversal training, conducted on day 6, the platform was located in the center of the opposite quadrant and submerged 1.0 cm inch below the surface of the water. Each animal was given four trials per day for 5 days as in the initial acquisition.

Reversal probe (PND 51)—A reversal probe trial was conducted approximately 24 hours after the final reversal training on day 12 of testing. The platform was removed from the pool and animals were allowed to swim for 90 s and time in the platform quadrant and crossings at the former platform location were recorded.

Data Analysis—Recorded data were analyzed through Ethovision software. For place navigation and reversal training, the latency to reach the platform and the swim speed for each animal were recorded. For all probe trials duration of time spent in the previous platform quadrant was recorded.

#### Marble Burying

On PND 54, subjects were placed in a standard Plexiglas test cage (30.5 cm x 35.6 cm x 20.3 cm) with a 5 cm deep layer of cobb bedding, and allowed to explore freely for 10 minutes. The subject was then placed in a transfer cage as 18 marbles (1.3 cm diameter, red) were placed on the bedding surface in a 3 x 6 pattern. The subject was then placed in the test cage and allowed to re-explore for 10 minutes while being video recorded by a Sony HDRCX240/B Video Camera with 2.7-Inch LCD (Black) fixed to a tripod. After 10 minutes, the subject was removed from the test cage and the number of marbles buried by at least 2/3 were counted. Videos were then scored using Observer XT 12 (Observer Version XT12, Noldus information Technology, the Netherlands) for marble interactions and digging behaviors. The full ethogram of behaviors scored is outlined in Table 3.

**Table 3:** Marble Burying Ethogram.

Behavior	Description
Exploration	Rat is actively exploring the arena for at least 1 second (i.e. sniffing bedding, walls, or water spout, rearing, etc.)
Marble Interaction	Rat rolls, pushes, lifts, or sniffs marble for at least 1 second.
Self Grooming	Rat displays self-grooming for at least 1 second.
Inactive	Rat sits or is hunched over for at least 1 second.
Digging	Rat displays digging or burrowing for at least 1 second.

### Statistical analyses

We compared anxiety-like behaviors from the standard elevated plus-maze between strains with a Mann-Whitney U test. A Wilcoxon signed rank test was used to analyze 3-chambered social approach data. Within each strain, we compared time spent in the chamber with the stimulus rat to time spent in the chamber with the novel object. Similarly, times in proximity to the stimulus rat versus times in proximity to the novel object, were compared within each strain, as modified by previous description in mice[61]. We tested for differences in the number of chamber entries during social approach between strains with two sample t-tests. For the open field data, total distance, horizontal activity, vertical activity, or center time were analyzed with a linear mixed effect model with an autoregressive covariance structure to account for correlated measurements over time. We first modeled trends over time within each strain and secondly compared strains by including strain as a predictor as well as an interaction term between strain and time. PPI data were compared within strains and between strains using a repeated measures ANOVA. For the reciprocal social interaction test, behavioral parameters were analyzed with Wilcoxon signed rank tests to compare strain differences. We focused on a limited number of salient behaviors to limit the possibility of type I error. Social interaction durations included: (i) social proximity, (ii) social exploration, (iii) social play. Nonsocial behaviors included: (i) cage exploration and (ii) self-grooming. The frequency of nape attacks initiated and received by the focal subjects was also analyzed. Behaviors outlined in the Morris Water maze were compared between strains using a repeated measures ANOVA. Behaviors outlined in the marble-burying test ethogram were analyzed with a Mann-Whitney U test for comparison between strains. We ran Kolgomorov-Smirnov tests to determine if data were normally distributed. In the cases where the assumption of normal distribution was rejected, we ran nonparametric analyses.

## Results

### Elevated Plus Maze

**Fig 1:**
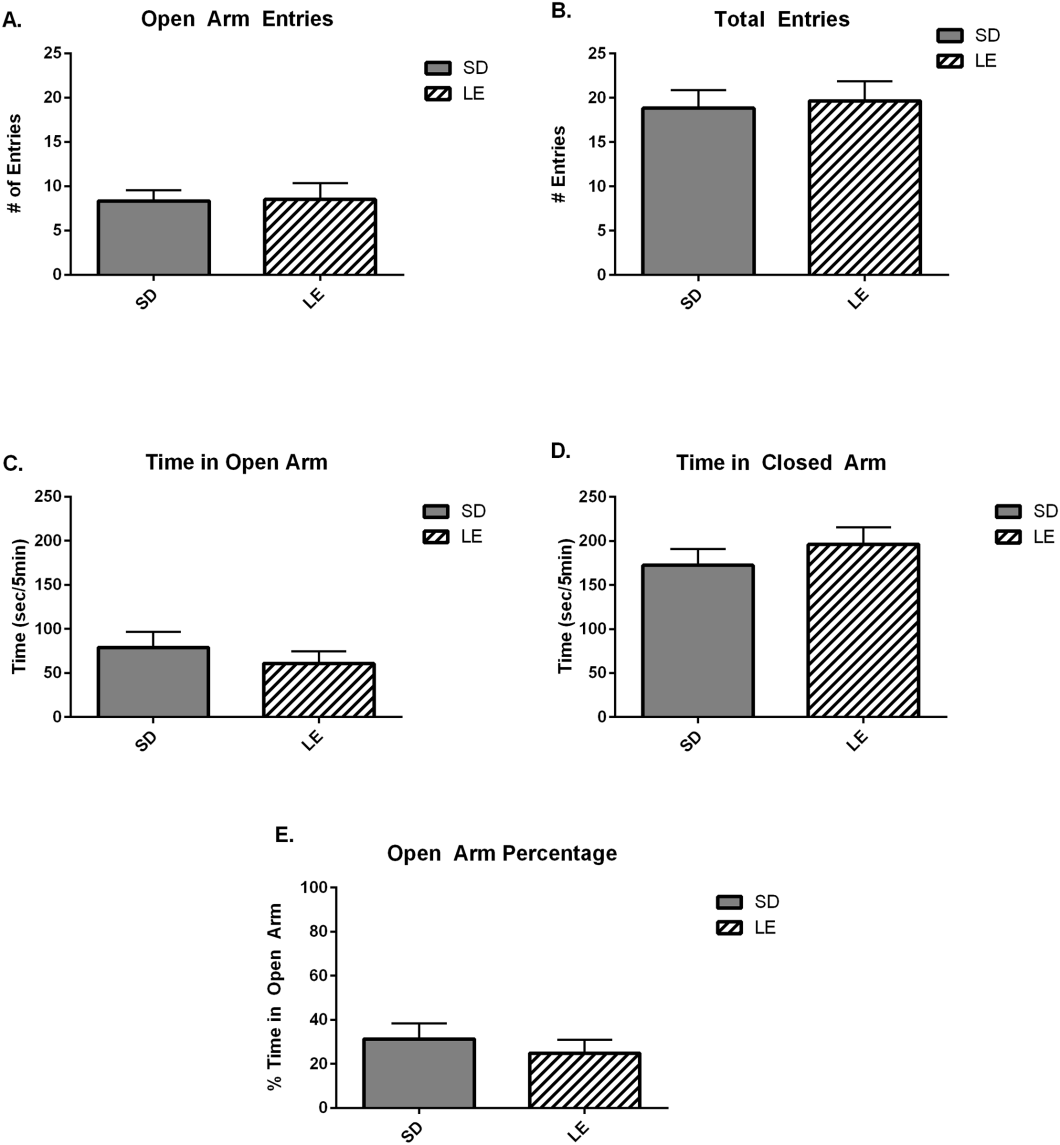
SD and LE rats show similar performance in elevated-plus maze. A) There were no strain differences in number of entries onto the open arms and no significant differences in total entries on both the open and closed arms of the maze (Panel B). Additionally, there were no significant differences between strains in duration of time spent on either open or closed arms (Panel C and D) or percentage of time on the open arms (Panel E).

Fig 1 illustrates the absence of anxiolytic-like or anxiogenic-like behavioral responses assessed using the standard elevated plus-maze. No strain differences were observed in the number of entries onto the open arms (Panel A, U = 68.00, *p =* 0.84) or the number of total entries on both the open and closed arms of the maze (Panel B, U = 51.50, *p =* 0.24). Additionally, there were no significant differences in time spent in the open arm or the closed arm (Panel C, U = 52.00, *p* = 0.27; Panel D, U = 55.00, *p* = 0.35) and no strain differences were observed in the percentage of time spent in the open arm (Panel E, U = 52.00, *p* = 0.27).

### Social Approach

**Fig 2:**
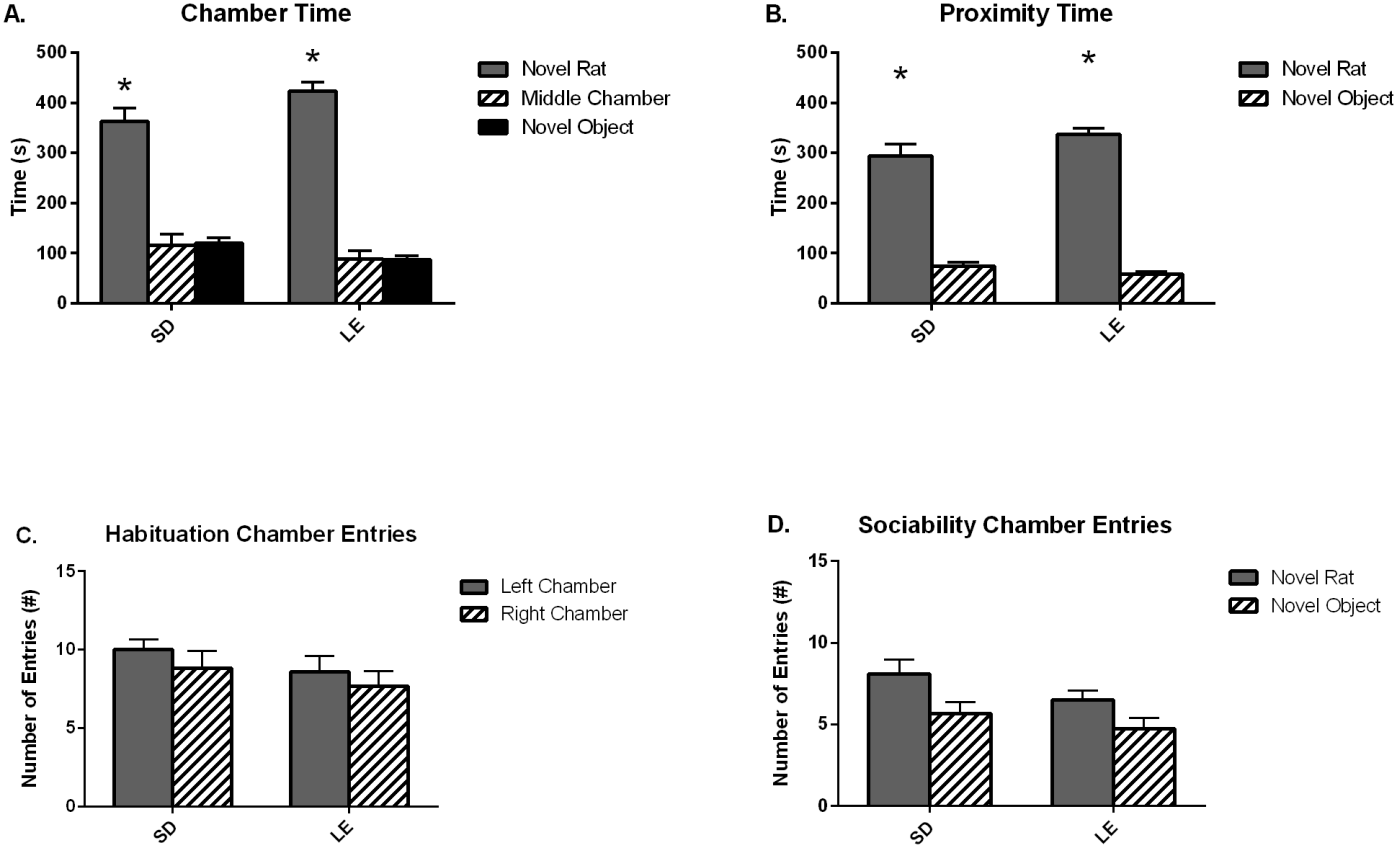
Both SD and LE strains exhibit high sociability in a three-chambered social approach task. A) Juvenile Sprague Dawley (SD) and Long Evans (LE) rats displayed sociability defined as spending more time in the chamber with the stimulus rat than in the chamber with the novel object and B) spending more time in the immediate proximity of the stimulus rat than the novel object. Both strains exhibited general exploratory activity throughout the apparatus as evidenced in the number of side chamber entries during the sociability and habituation phases (Panel C and D). Data shown are mean (+SEM) for each strain in a ten minute test.

Fig 2 illustrates high sociability scores from the automated 3-chambered social approach task in SD and LE rats. Significant sociability was detected in SD and LE, as expected by these strains of social rodent species (Panel A, SD: Z = -2.98, *p* < 0.01; LE: Z = -2.67, *p* = 0.44). SD and LE exhibit significantly more time in the chamber with the stimulus rat than time in the chamber with the novel object, providing strong evidence for expected sociability in both strains. Similar to the chamber time parameter of sociability, SD and LE subject rats also displayed significant sociability as indexed by time spent in the immediate proximity (within 2cm) of the cup containing the stimulus rat as compared to time spent in the immediate proximity of the empty cup (Panel B, SD: Z = -2.82, *p* < 0.01; LE: Z = -3.06, *p* < 0.01). There were no differences between strains in the number of entries into the two side chambers during the initial 10 min habituation phase ((Panel C, Left Chamber: t_(22)_ = 1.16, *p* = 0.26; Right Chamber: t_(22)_ = 0.79, *p* = 0.44) or during the sociability phase (Panel D, Social Chamber: t_(22)_ = 1.84, *p* = 0.08; Nonsocial Chamber: t_(22)_ = -0.65, *p* = 0.52), indicating that general exploratory activity did not differ between strains during the social approach assay. These data for exploratory activity are corroborated by the open field results.

### Open Field

**Fig 3:**
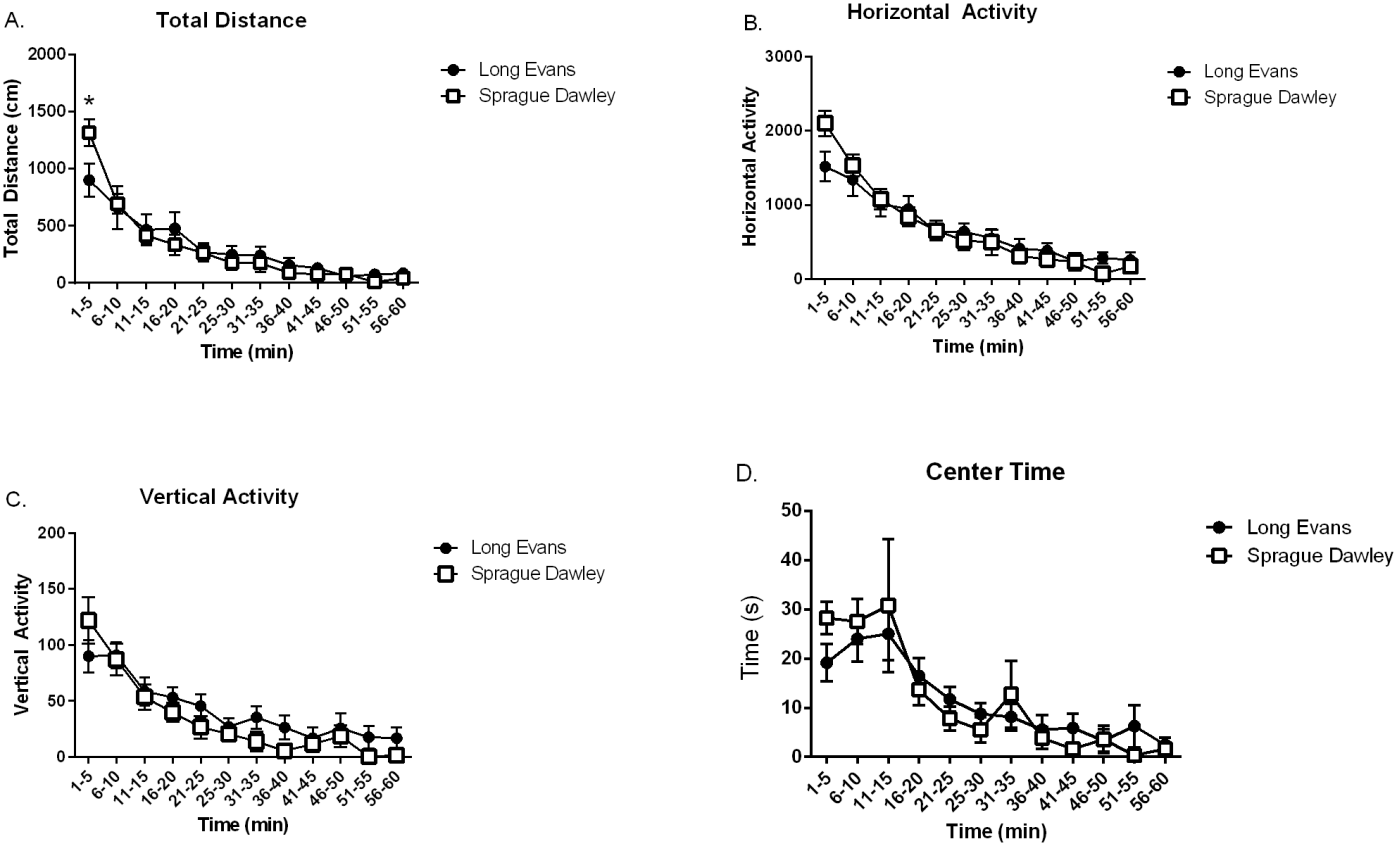
Both strains exhibited similar exploratory locomotion patterns in an open field task. Both SD and LE strains showed a decline in horizontal activity and total distance over the course of the 60 minute task as expected (Panel A and B). Similarly, both strains showed a decline in vertical activity and a decrease in time spent in the center of the arena over the 60 minute time course (Panel C and D). There were no significant strain differences.

Fig 3 illustrates the activity curves on four parameters assessed in the open field arena for exploratory locomotion in SD and LE rats. Across the 60 minute session, the time course for total distance travelled and horizontal activity, in both SD and LE declined as expected, reflecting habituation to the novel open field (Main effect of time: Panel A, SD: F _(11,90)_ = 25.20, *p* < 0.01; LE: F _(11,101)_ = 4.85, *p* < 0.01; Panel B, SD: F _(11, 90)_ = 14.61, *p* < 0.01; LE: F _(11, 93)_ = 5.02, *p* < 0.01). Total distance and horizontal activity scores were not different between both strains (Main effect of strain: Panel A, F _(1, 30)_ = 0.009, *p* = *0*.92; Panel B, F _(1, 35)_ = 0.001, *p* = *0*.97). A significant interaction between strain and total distance traveled was revealed (Panel A, F _(11,195)_ = 2.64, *p* = 0.04). The two strains differed in total distance traveled during the first 5 minute time interval but not during subsequent time intervals (min 1–5, *p* < 0.01; min 6–10, *p* = 0.66; min 11–15, *p* = 0.97; min 16–20, *p* = 0.55; min 21–25, *p* = 0.82; min 26–30, *p* = 0.84; min 31–35, *p* = 0.87; min 36–40, *p* = 0.86; min 41–45, *p* = 0.93; min 46–50, *p* = 0.58; min 51–55, *p* = 0.78; min 56–60, *p* = 0.74). Results for horizontal activity revealed no significant interaction between strain and horizontal activity overall (Panel B, F _(11, 183)_ = 1.52, *p* = 0.13). Vertical activity over the 60 minute test period declined as expected in both SD and LE rat strains (Main effect of time: Panel C, SD: F _(11, 69)_ = 10.17, *p* = 0.00; LE: F _(11, 80)_ = 4.32, *p* = 0.00). Vertical activity scores were not different between SD and LE (Main effect of strain: Panel C, F _(1, 45)_ = 1.73, *p* = 0.20) and no significant interaction between strain and vertical activity was observed (Panel C, F _(11, 151)_ = 1.13, *p* = *0*.35). Time in the center of the arena did not differ between SD and LE (Main effect of strain: Panel D, F _(1, 80)_ = 0.00, *p* = 0.99), nor was there a significant strain by time interval interaction for center time (F _(11, 157)_ = 0.59, *p* = 0.84). Time spent in the center of the test arena decreased over time for SD and LE (Main effect of time: Panel D, SD: F _(11, 78)_ = 4.46, *p* < 0.01; LE: F _(11, 80)_ = 3.54, *p* < 0.01).

### Prepulse Inhibition

**Fig 4:**
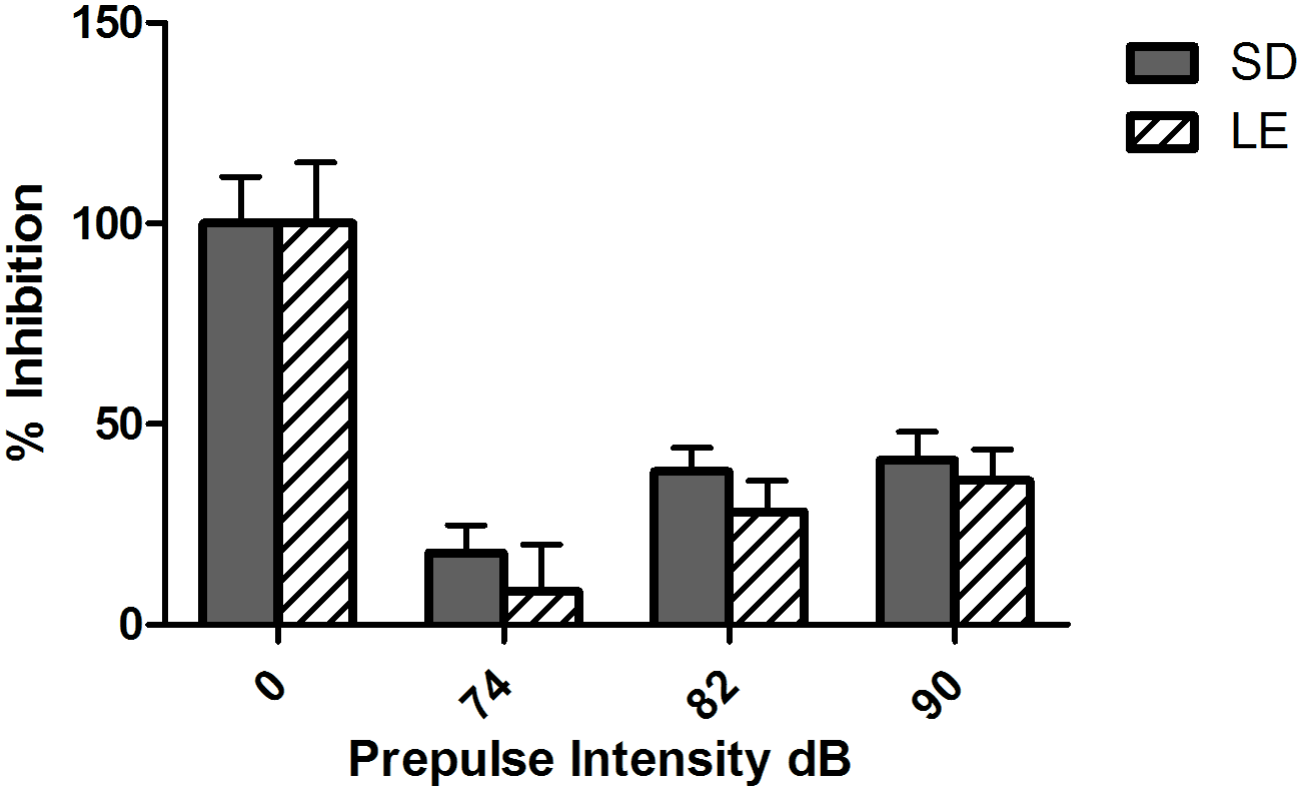
Prepulse Inhibition shows no significant strain differences in SD and LE rats. No significant effects of strain on inhibition of the startle response by prepulses were observed between SD and LE nor was an interaction between strain and prepulse intensity observed.

Normal prepulse inhibition of acoustic startle seen in SD and LE indicates intact sensorimotor gating. As seen in Fig 4, prepulse decibel intensity affected startle response inhibition in SD (F (_(3,33_) = 69.21, *p* < 0.01) and LE (F _(3,33)_ = 39.90, *p* < .01), as prepulse intensity increased sensorimotor reactivity was more inhibited, as expected. No significant effects of strain on inhibition of the startle response by prepulses were observed between SD and LE (Fig 4, F _(1, 22)_ = 1.07, *p* = 0.31) nor was an interaction between strain and prepulse intensity observed (Fig 4, F _(3, 66)_ = 0.90, *p* = 0.45).

### Social Dyads

**Fig 5:**
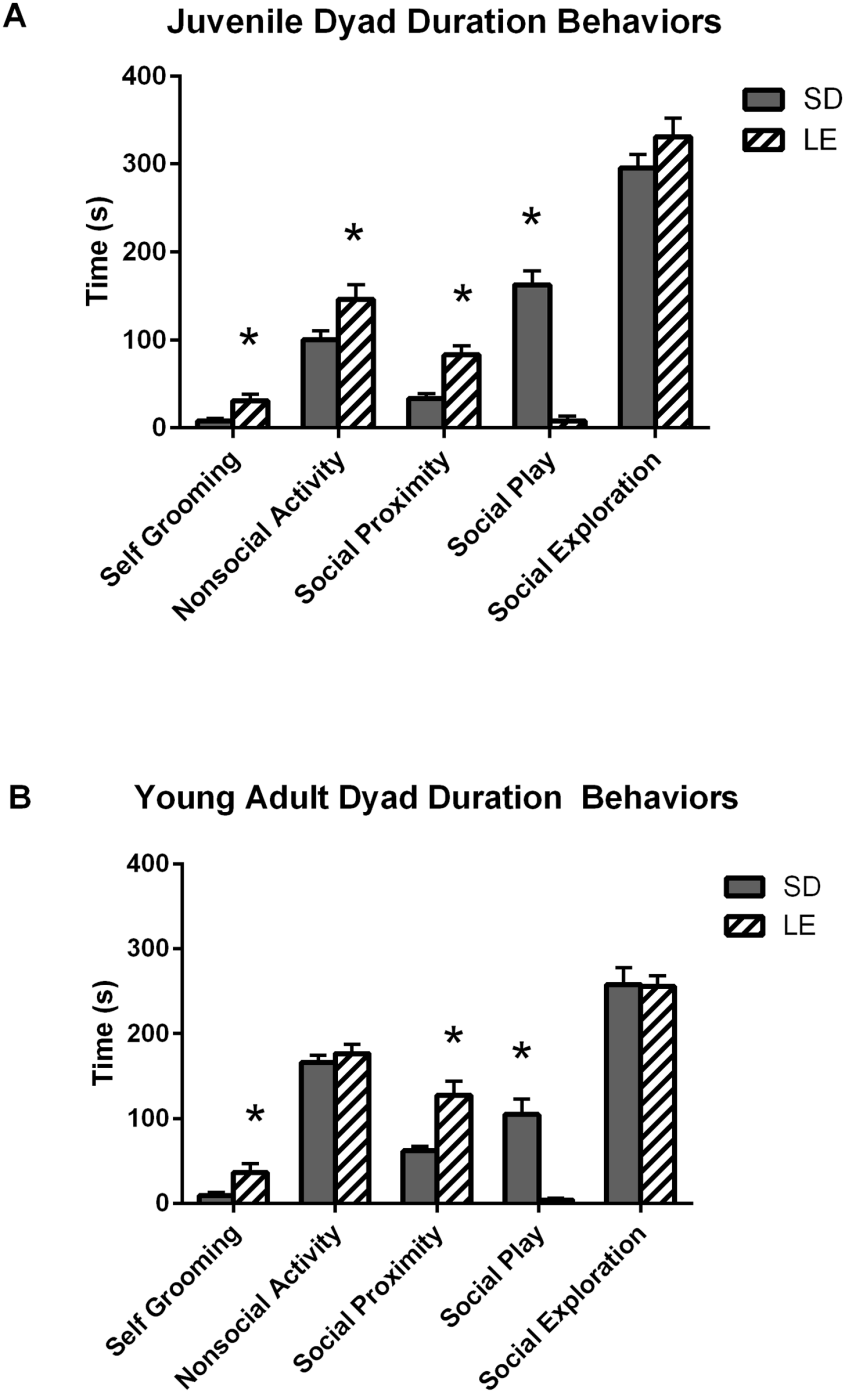
Significant strain differences in duration of social behaviors at both juvenile and adolescent (young adult) ages during a social dyad task. Two unfamiliar male rats engaged in social interactions for both SD and LE strains at both juvenile and adolescent time points. A) As juveniles (ages 36–37), LE rats spent more time self-grooming (*p* < 0.01), in nonsocial activity (*p* = 0.02), or in social proximity than did SD rats of the same age (*p* < 0.01). SD rats exhibited significantly more social play behavior than LE rats (*p* < 0.01). B) As young adults (ages 55–56), LE rats showed more time self-grooming (*p* < 0.01) or in a state of social proximity (*p* < 0.01) than SD rats while SD rats exhibited more time in a state of social play than LE rats (*p* < 0.01). Data shown are mean (+SEM) for a 10 minute task.

Duration. Fig 5 illustrates the duration of social and nonsocial behaviors during dyadic interactions with a stimulus, but otherwise naïve, social partner at juvenile (P36-37; Panel A) and young adult (P55-56; Panel B) ages. LE rats spent more time self-grooming at both the juvenile (Panel A, U = 12.00, *p* < 0.01) and young adult time points (Panel B, U = 20.00, *p* < 0.01). Juvenile LE rats spent more time in nonsocial activity than did the SD rats (Panel A, U = 29.00, *p* = 0.02), though significant differences were not detected for nonsocial activity at the young adult time point (Panel B, U = 55.00, *p* = 0.53). Although no strain differences were observed in the time spent in social exploration (i.e., sniffing, following, grooming or contacting) with the stimulus partner at juvenile (Panel A, U = 48.00, *p* = 0.29) or young adult (Panel B, U = 61.00, *p* = 0.79) time points, pronounced differences were detected in other social behaviors. Compared to LE rats, the SD rats spent more time engaged in social play at both juvenile (Panel A, U = 0.00, *p* = 0.00) and young adult (Panel B, U = 0.00, *p* = 0.00) time points. In contrast, the LE rats spent more time in social proximity (within 2 cm, but not interacting) with stimulus partners at juvenile (Panel A, U = 11.00, *p* < 0.01) and young adult (Panel B,U = 11.00, *p* < 0.01) time points.

**Fig 6:**
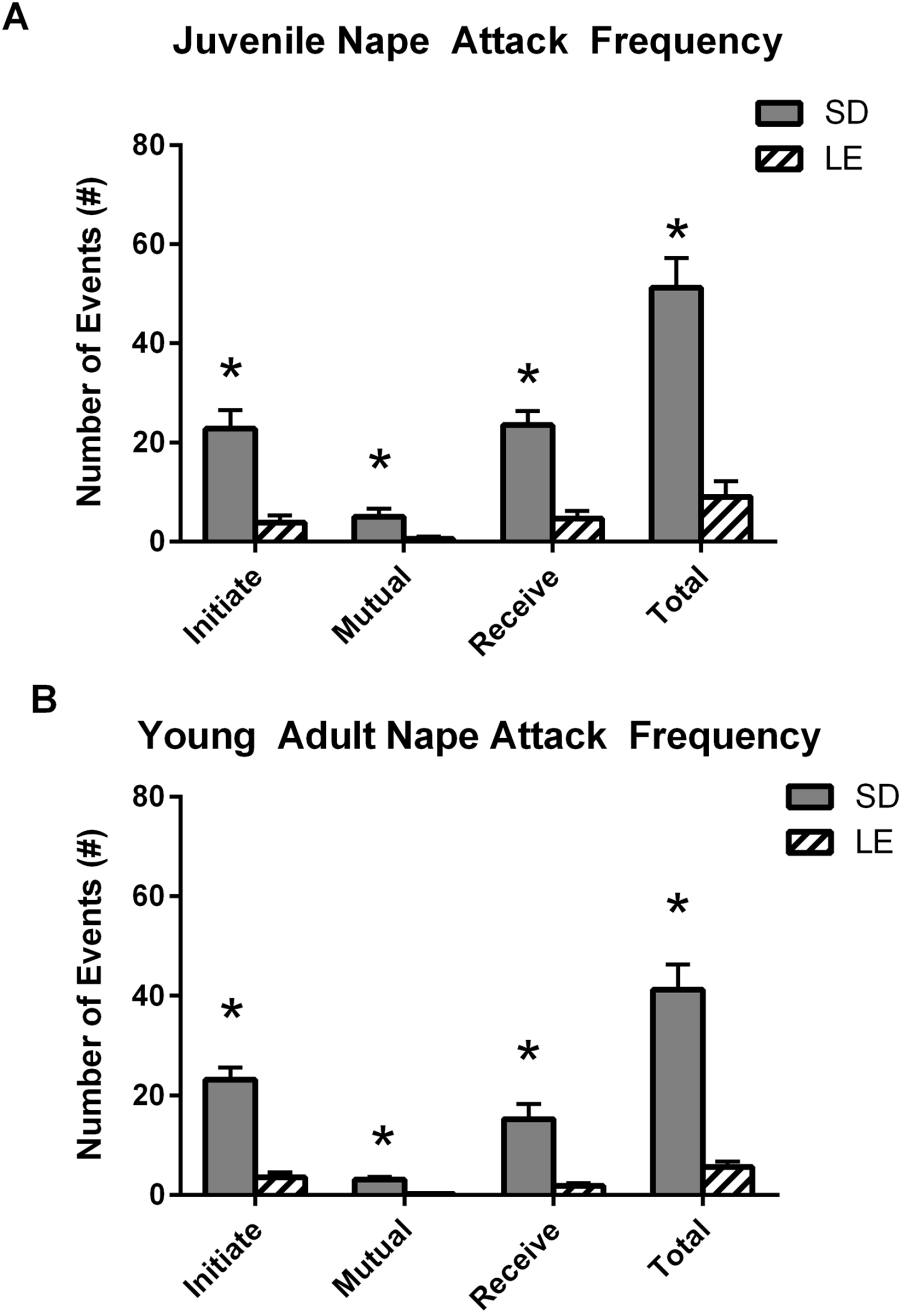
Significant strain differences in number of nape attacks at juvenile and young adult time points in SD and LE rats during a social dyad task. A) SD rats show significantly more social play behavior, expressed in nape attacks, than LE rats at ages PND 36–37 and B) at ages PND 55-56Data shown are mean (+SEM) for a 10 minute task.

Frequency. Fig 6 illustrates the frequency of nape attacks initiated and received at juvenile (P36-37; Panel A) and young adult (P55-56; Panel B) time points. Data represent the total occurrences of each behavior initiated and received by the focal rat during the 10 minute social dyad. Compared to LE rats, the SD rats more frequently initiated nape attacks at both the juvenile (Panel A, U = 10.00, *p* < 0.01) and young adult (Panel B, U = 2.00, *p* < 0.01) time points. SD rats also received more nape attacks at both the juvenile (Panel A, U = 3.50, *p* < 0.01) and young adult (Panel B, U = 3.00, *p* < 0.01) time points. Significant strain differences were also found for mutual nape attacks, in which the focal and partner rat exchanged mutual nape attacks in rapid succession at juvenile (Panel A, U = 20.00, *p* < 0.01) and young adult (Panel B, U = 0.00, *p* < 0.01) time points. Frequency data are summarized in Table 4.

**Table 4:** Social Dyads at Juvenile and Young Adult time points—Frequency of Nape Attacks.

Behavior	Juvenile			Young Adult		
Nape Attack	Mean	SEM	p value	Mean	SEM	p value
**Initiate**						
LE	3.818	1.494	*p* < .01	3.909	1.031	*p* < 0.01
SD	22.750	3.834		23.167	2.449	
**Mutual**						
LE	0.545	0.455	*p* = 0.02	0.182	0.122	*p* < 0.01
SD	5.000	1.656		3.091	0.530	
**Receive**						
LE	5.201	1.568	*p <* 0.01	1.909	0.563	*p* < 0.01
SD	10.041	2.899		15.250	3.058	
**Total**						
LE	9.000	3.188	*p* < 0.01	6.000	1.104	*p* < 0.01
SD	51.250	6.004		41.250	5.027	

### Morris Water Maze

**Fig 7:**
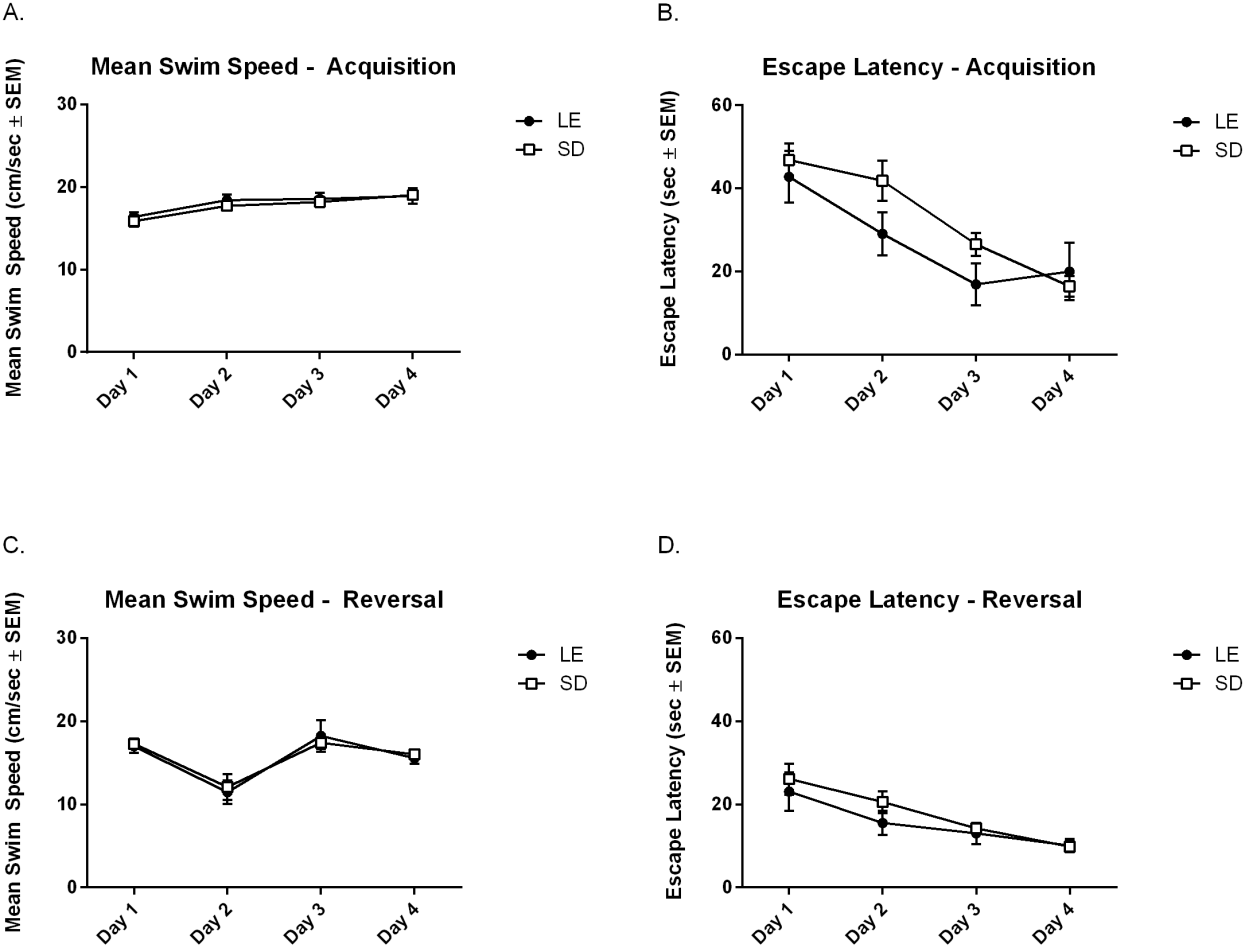
There were no significant strain differences in mean swim speed, escape latency, or percentage of time spent in the quadrants for both acquisition and reversal trials of the Morris Water Maze task.

For the Morris Water Maze, during acquisition there were no statistically significant strain differences in swim speed (Fig 7 Panel A, F_(1,22)_ = 0.27, *p* = 0.61), latency to find the escape platform (Fig 7 Panel B, F_(1,22)_ = 1.03, *p* = 0.32), and no significant strain differences during the probe trial (data not shown). During reversal learning animals readily learned a new platform location. However, there were again no significant strain differences in swim speed (Panel C, F_(1,22)_ = 0.02, *p* = 0.88), escape latency (Panel D, F_(1,22)_ = 0.537, *p* = 0.47), and no strain differences in performance during the probe trial (data not shown).

### Marble Burying

**Fig 8:**
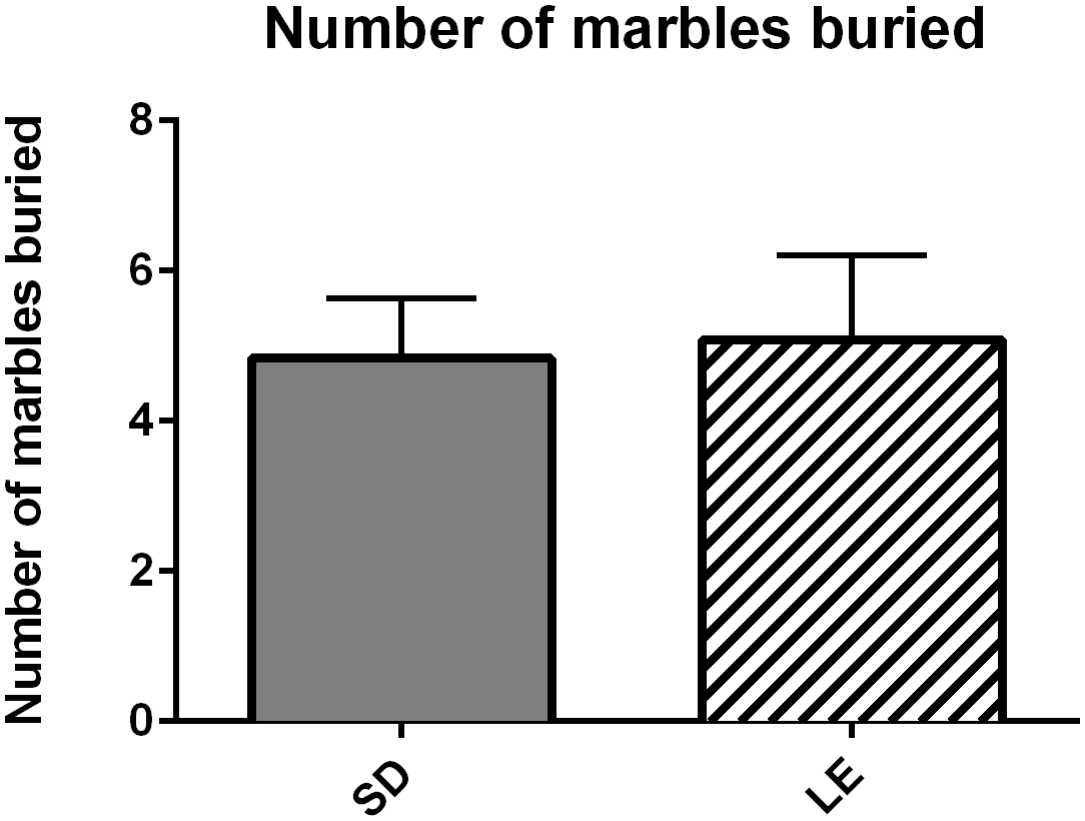
No significant strain differences in number of marbles buried. Data shown are mean (+SEM) for a 10 minute task.

**Fig 9:**
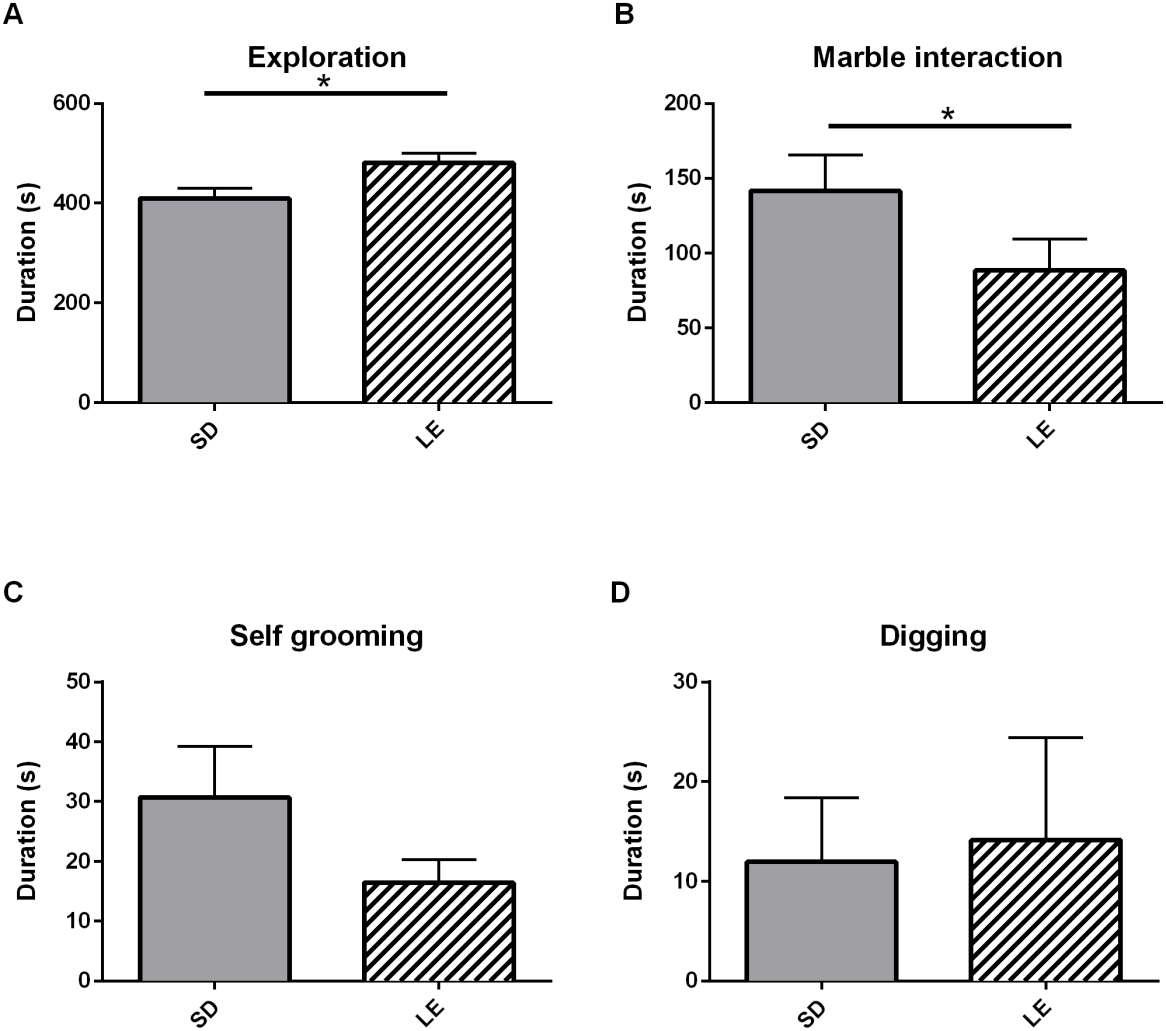
Manually scored behaviors during marble burying. A) SD rats spent less time exploring (rearing plus active exploration of the arena) than the LE rats. B) SD rats also spent more time interacting with the marbles than the LE rats though there were no strain differences in the other parameters of C) self-grooming and D) digging. Data presented are mean (+SEM).

There were no strain differences in total number of marbles buried during the 10 minute task (Fig 8, U = 71.50, *p* = 0.98). SD rats did spend less time exploring the marble burying chamber than the LE rats (Fig 9 Panel A, U = 25.00, *p* = 0.01), and spent more time interacting with the marble (Fig 9 Panel B, U = 33.00, *p* = 0.04).

## Discussion

The potential to explore genetic, environmental and pharmacological manipulations in a species that exhibits a complex social repertoire makes the laboratory rat an attractive model for preclinical ASD research. As an initial step in developing rat models of ASD, we first compared albino Sprague-Dawley (SD) and hooded Long-Evans (LE) rat strains using behavioral assays commonly used in mouse ASD models [7]. During the juvenile through young adult period (postnatal day 26–56) the two strains were comparable on measures of activity, sensorimotor gating, anxiety, repetitive behaviors, and learning and memory. Given that deficits in social functioning are a hallmark feature of ASD, we focused our efforts on measures of social behavior, including the automated three-chamber social approach assay and manually-scored reciprocal dyadic social interactions. Both LE and SD strains demonstrated sociability in the automated 3-chamber task, as defined by spending more time in the chamber with a constrained stimulus than in an identical chamber without a stimulus partner. In contrast, pronounced strain differences were detected during dyadic interactions with an unconstrained stimulus partner. The SD rats in the present study spent significantly more time than LE rats in social play behavior at both juvenile (PND 35) and young adult (PND 56) developmental ages. Nape attacks, a characteristic feature of rat play behavior, were more frequently initiated and received by SD compared to LE rats. Strain-specific social behaviors have been previously reported in adolescent mice [62,63] and rats [31,32] and are not unexpected given that animals from different genetic background exhibit variations in physiology, neuroanatomy and other behavioral parameters [64–70]. However, given that play behavior in LE rats has been well documented in other laboratories [28], it is plausible that subtle changes in the testing protocols may exacerbate strain-specific differences in social behavior. The implications of these findings are discussed within the broader context of utilizing rats in preclinical ASD research.

As ASD is a behaviorally defined disorder, preclinical models rely on behavioral phenotyping tools to evaluate the distinctive clinical features of ASD in a non-human species. Much effort has focused on developing behavioral assays relevant to the first DSM-V diagnostic criterion defined as, “persistent deficits in social communication and social interaction across multiple contexts”. Individuals diagnosed with ASD may exhibit deficits in (i) social-emotional reciprocity, ranging from abnormal social approach and failure to initiate or respond to social interactions, (ii) nonverbal communicative behaviors used for social interaction, ranging from poorly integrated verbal and nonverbal communication to a total lack of facial expressions and nonverbal communication and (iii) deficits in developing, maintaining, and understanding relationships, including difficulties adjusting behavior to suit various social contexts and/or an absence of interest in peers [1]. There are, however, challenges in modeling these complex behavioral manifestations of ASD in non-human species. Automated behavioral assays, such as the commonly used three-chamber social approach, provide a relatively simple assay of social interest as determined by the time the experimental animal spends near another animal that is confined to a small cage [47]. In mice, for example, the species-typical response to an unfamiliar conspecific is to approach and investigate, and decreased time spent investigating a stimulus animal is operationally defined as diminished sociability [25,40,43,71]. Here we described a version of the three chamber apparatus modified for use in rats and demonstrate that both SD and LE strains demonstrate “sociability” as defined as spending more time in the chamber with the stimulus age/sex/strain/weight matched rat than in the chamber with the novel object. Although the three-chambered social approach test is commonly used as a first line screening assay for autism like phenotypes in mouse models [47], there are perhaps limitations in relying heavily on a social assay that does not allow animals to freely engage in reciprocal social behavior. The measures of sociability generated in the three chamber assay have been interpreted as an all-or-none result that should be restricted to comparison with a single experimental group, rather than a comparison between groups [7]. Although this behavioral task has been useful in mouse models of ASD, it may not be sensitive to more subtle impairments in social behavior that may be detected in rats, nonhuman primates, and other species that engage in reciprocal social behaviors, such as juvenile play [50]. Given that rats are generally considered to have a more extensive social repertoire than mice, the rat may provide an opportunity to augment simple automated procedures by focusing on more complex ASD-relevant social interaction assays.

Deficits in social play are a prominent feature of ASD [72,73] that can be measured in preclinical animal models by measuring the frequency and duration of species-typical play behaviors. Play fighting is the most common form of play behavior in rats [29,56] and is initiated when one partner uses their snout to nuzzle the nape of the neck of the other animal and the partner, in turn, defends their nape from such attacks by rotating to its dorsal surface or evading the attacker. Rat play behavior typically emerges in the week preceding weaning (approximately PND 16–17), peaks between PND 30–35 and declines thereafter [60]. Dyadic interactions were carried out within the peak of play behavior at juvenile (PND 35) and young adult (PND 56) time points. We compared the amount of time the strains engaged in social play (i.e., pouncing/nape attack, pinning, boxing, wrestling and chasing), social exploration (i.e., sniffing, following, non-play contacting, grooming), social proximity (i.e., within 2cm of the partner, but not directly interacting), nonsocial activity (i.e., exploring the cage alone) or self-grooming) (See Fig 5). We then quantified the frequency of nape attacks initiated and received by the focal subject to provide a more fine-grained assessment of reciprocal play interactions (See Table 4). SD and LE strains spent comparable amounts of time exploring stimulus partners, but there were pronounced strain differences for play-related behaviors. At PND 35, juvenile SD rats spent more time playing and more frequently initiated and received nape attacks than did LE rats. In contrast, juvenile LE rats spent more time in proximity to, but not interacting with the partner. We then evaluated dyadic interactions with a stimulus partner at the early stages of adulthood (PND 56). The young adult SD rats continued to spend significantly more time playing than did LE rats, and also initiated and received nape attacks more frequently. Again, LE rats spent more time in proximity to, but not interacting with, the stimulus partner. Although the amount of time SD rats played decreased from 25% in juveniles to 18% in young adults, it is important to note that relatively high levels of play continued well into the early young adult time period. Given that the hallmark social deficits in ASD emerge very early in development, the protracted period of reciprocal social interaction documented in the rat will provide a valuable test platform for ASD-focused research. However, choices in strain, behavioral assays and testing environment are likely to have a profound effect on outcome measures.

Young SD and LE rats in the present study did not differ on measures of anxiety, sensorimotor processing or exploration, suggesting that the low levels of play produced by the LE rats were not due to other behavioral differences that may indirectly impact social interactions. It is, however, plausible that our dyad testing protocol may have inadvertently favored play conditions for the SD strain. Numerous studies have identified environmental factors that influence rat social behavior, including the length of social isolation preceding the test, familiarity with the testing environment and properties of the testing environment, including cage size, lighting, bedding [74–77]. Taking these factors into consideration, we developed a dyad protocol that could be used to reliably quantify reciprocal social interactions with a relatively high throughput capacity. Our goal was to develop a relatively high throughput social assay drawing from the rich literature on rat social behavior, which often utilizes frame-by-frame scoring to capture the nuances of rat social interactions [26–30]. We made several changes to standard rat play observational protocols that would allow this assay to be integrated into a broader phenotyping battery that includes measures of repetitive behaviors and associated ASD symptoms, similar to more established mouse ASD model phenotyping tools [7]. For example, although social isolation amplifies the frequency of play behaviors in juvenile rats, and is commonly used to elicit play behaviors [55,60,71,78], we opted to conduct the social dyads without an extended isolation period. Given that ASD is characterized by persistent deficits on social communication and interaction that cause clinically significant impairments in social functioning [79], baseline measure of social play (not prompted by social isolation) may be more directly relevant. Moreover, previous studies in rats indicate that different forms of social behavior are differentially sensitive to social deprivation across ontogeny [80], thus we opted to avoid extended isolation periods in order to quantify baseline play behaviors at both juvenile and young adult time points, without the influence of prolonged isolation. The rats in the present study were only isolated for 10 minutes prior to the dyad, during habituation to the test cages, and then observed interacting with the stimulus partner under dim lighting. We also excluded bedding from the test arena to facilitate the acquisition of ultrasonic vocalizations in future studies using this same paradigm [81]. Although the levels of play reported in our study are not directly comparable to studies that utilize extended isolation periods [32,42], our results are consistent with previous studies suggesting that SD rats exhibit robust play levels in a variety of testing conditions paradigms [42,61,80]. In contrast, paradigms optimized for eliciting play behavior in LE rats utilize 24 hours of social isolation prior to testing, repeated habituation to the test cage, testing in complete darkness and the presence of bedding [27,41,57,82]. It is unclear which of these factors is most critical for eliciting play behavior in LE rats, though bedding may be particularly important for LE rats (and other strains) that utilize the “turn and face” defensive play behavior that is more likely to include complete rotation and supine defense postures [32]. In contrast, the SD rats spent more time engaged in play behavior, consisting of nape attacks embedded in prolonged bouts of evasive defensive maneuvers characteristic of wild type and SD rats (i.e., running, leaping or pivoting away from the other rat) [31–33]. The evasive play style of SD rats offer robust levels of play behavior, suitable for analysis of frequency and duration outcomes, while the turn and face play style of LE rats may provide more opportunity to quantify the sequence of face to face play interactions.

SD and LE rats were also compared using behavioral phenotyping tools relevant to the second ASD DSM-V diagnostic criterion, “restricted, repetitive patterns of behavior, interests, or activities”. Although the untreated LE rats engaged in more self-grooming than SD rats during social dyads, the overall levels of self-grooming were relatively low (approximately 5% of the time) and would not be interpreted as excessive self-grooming that is characteristic of other ASD rodent models [59,83]. Quantification of marble-burying has been proposed as a measure of repetitive digging behavior and/or anxiety relevant behaviors in rodents [84–86]. In mice, we know that the test is sensitive to strain, brain lesion and pharmacological treatment [87]. Both antidepressant drugs and antianxiety drugs have been shown to reduce marble burying behavior, leading to the test being widely used in the field of anxiety research [88]. However, other groups use it as a measure of perseverative digging behavior rather than novelty-induced anxiety [89,90]. There were no significant differences in the number of marbles buried by SD or LE rats. However, our pilot work indicated that rats can pick up and play with marbles more readily than mice and many marbles become partially covered with bedding due to movement of the rat around the cage. We therefore used archived videos to measure the amount of time devoted to digging and interacting with the marbles rather than relying solely on a still image at the end of the experiment. Although there were no differences in the number of marbles buried, SD rats did spend more time interacting with the marbles. Importantly, we did not observe the characteristic defensive burying response that has been described in the mouse literature, suggesting that digging and burying behaviors differ across muroid species [91].

The data presented here demonstrate the potential for utilizing the laboratory rat in preclinical ASD research, and also highlight important considerations in designing and interpreting ASD-relevant behavioral assays for use in rat models. This is critical information for investigators interested in developing rat models of ASD to consider. The lack of play behaviors produced by the LE rats in our dyad testing paradigm indicates that changes in husbandry practices, isolation time, and experimental conditions can exacerbate strain-typical patterns of social interactions and potentially influence the interpretation of ASD related behavioral outcomes. Moreover, the absence of strain differences detected on the three chamber social approach test suggests that assays that do not allow animals to freely interact are unlikely to fully capture the complexities of social interactions in species that exhibit complex, reciprocal social interactions. We suggest that ASD focused rat models should also include social behavior assays that allow the animals to physically interact with one another in a semi-naturalistic environment, preferably across multiple developmental time points. Future efforts should further capitalize on the complex social behavior of the rat model by incorporating other aspects of social communication, such as ultrasonic vocalizations [11,92] as well as assays of more complex social processing, including social reward and empathy [93–95], to maximize relevance to ASD.

## Supporting Information

S1 File Strain Comparison Stats (2). This excel workbook compiles the raw data from the behavioral assays.
(XLSX)
